# Progressive Multi-Scale Vision Transformer for Facial Action Unit Detection

**DOI:** 10.3389/fnbot.2021.824592

**Published:** 2022-01-12

**Authors:** Chongwen Wang, Zicheng Wang

**Affiliations:** School of Computer Science, Beijing Institute of Technology, Beijing, China

**Keywords:** affective computing, facial action unit recognition, multi-scale transformer, self-attention, cross-attention

## Abstract

Facial action unit (AU) detection is an important task in affective computing and has attracted extensive attention in the field of computer vision and artificial intelligence. Previous studies for AU detection usually encode complex regional feature representations with manually defined facial landmarks and learn to model the relationships among AUs *via* graph neural network. Albeit some progress has been achieved, it is still tedious for existing methods to capture the exclusive and concurrent relationships among different combinations of the facial AUs. To circumvent this issue, we proposed a new progressive multi-scale vision transformer (PMVT) to capture the complex relationships among different AUs for the wide range of expressions in a data-driven fashion. PMVT is based on the multi-scale self-attention mechanism that can flexibly attend to a sequence of image patches to encode the critical cues for AUs. Compared with previous AU detection methods, the benefits of PMVT are 2-fold: (i) PMVT does not rely on manually defined facial landmarks to extract the regional representations, and (ii) PMVT is capable of encoding facial regions with adaptive receptive fields, thus facilitating representation of different AU flexibly. Experimental results show that PMVT improves the AU detection accuracy on the popular BP4D and DISFA datasets. Compared with other state-of-the-art AU detection methods, PMVT obtains consistent improvements. Visualization results show PMVT automatically perceives the discriminative facial regions for robust AU detection.

## 1. Introduction

Facial expression is a natural way for non-verbal communication in our daily life and can be considered as an intuitive illustration of human emotions and mental states. There are some popular facial expression topics categorized as discrete facial expression categories, facial micro-expression, and the Facial Action Coding System (FACS) (Ekman and Friesen, 1978). Among them, FACS is the most comprehensive, anatomical system for encoding expression. FACS defines a detailed set of about 30 atomic non-overlapping facial muscle actions, i.e., action units (AUs). Almost any anatomical facial muscle activity can be introduced *via* a combination of facial AUs. Automatic AU detection has drawn significant interest from computer scientists and psychologists over recent decades, as it holds promise to several practical applications (Bartlett et al., 2003; Zafar and Khan, 2014), such as human affect analysis, human-computer interaction, and pain estimation. Thus, a reliable AU detection system is of great importance for the analysis of fine-grained facial expressions.

In FACS, different AUs are tightly associated with different facial muscles. It actually means we can observe the active AUs from specific facial regions. For example, the raising of the inner corners of the eyebrows means activated AU1 (inner brow raiser). Lowering the inner corners of the brows corresponds to AU4 (brow lowerer). AU annotators are ofter unable to describe the precise location and the facial scope of the AUs due to the ambiguities of the AUs and individual differences. Actually, the manually defined local AU regions are ambiguous. Existing methods (Li et al., 2017a,b, 2018a,b; Corneanu et al., 2018; Shao et al., 2018; Jacob and Stenger, 2021) usually use artificially define rectangle local regions, or use adaptive attention masks to focus on the expected local facial representations. However, the rectangle local regions violate the actual appearance of the AUs. Moreover, several AUs are simultaneously correlated with multiple and fine-grained facial regions. The learned adaptive attention masks fail to perceive the correlations among different AUs. Therefore, it is critical to automatically learn the AU-adaptive local representations and perceive the dependencies of the facial AUs.

**Figure 1:**
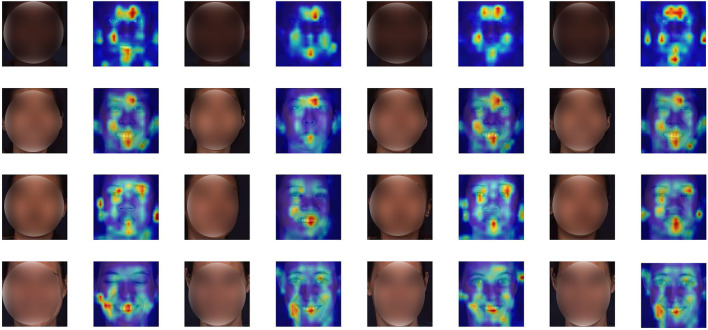
Attention maps of some faces. Our proposed PMVT is capable of capturing the AU-specific facial regions for different identities with diverse facial expressions.

To mitigate this issue, we introduce a new progressive multi-scale vision transformer (PMVT) to capture the complex relationships among different AUs for the wide range of facial expressions in a data-driven fashion. PMVT is based on the multi-scale self-attention mechanism that can flexibly attend to a sequence of image patches to encode the critical cues for AU detection. Currently, vision transformers (Dosovitskiy et al., 2020; Li et al., 2021) have shown promising performance across several vision tasks. The vision transformer models contain MSA mechanisms that can flexibly attend to a sequence of image patches to encode the dependencies of the image patches. The self-attention in the transformers has been shown to effectively learn global interactions and relations between distant object parts. A series of works on various tasks such as image segmentation (Jin et al., 2021), object detection (Carion et al., 2020), video representation learning (Girdhar et al., 2019; Fang et al., 2020) have verified the superiority of the vision transformer models. Inspired by CrossViT (Chen et al., 2021) that processes the input image tokens with two separate transformer branches, our proposed PMVT firstly uses the convolutional neural network (CNN) to encode the convolutional AU feature maps. Then PMVT obtains the multi-scale AU tokens with the small-patch and large-patch branches. The two branches receive different scale AU tokens and exchange semantic AU information *via* a cross attention mechanism. The self-/cross-attention mechanisms facilitate PMVT the content-dependent long-range interaction perceiving capabilities. Thus, PMVT can flexibly focus on the region-specific AU representations and encode the correlations among different AUs to enhance the discriminability of the AU representations. Figure 1 shows the attention maps of several faces. It is clear that PMVT is capable of focusing on the critical and AU-related facial regions for a wide range of identities and races. More facial examples and detailed explanations can be seen in section 4.2.1.

In summary, the contributions of this study are as follows:

1. We introduce a PMVT for facial AU detection. PMVT does not rely on manually defined facial landmarks to extract the regional AU representations.
2. To further enhance the discriminability of the facial expression representation, PMVT consists of separate transformer branches that receive the multi-scale AU tokens as input. PMVT is capable of encoding multi-scale facial AU representations and perceiving the correlations among different AUs to facilitate representing different AU flexibly.
3. Experimental results demonstrate the advantages of the proposed PMVT over other state-of-the-art AU detection methods on two popular AU datasets. Visualization results show that PMVT is superior in perceiving and capturing the AU-specific facial regions.

## 2. Related Work

We focus on the previous studies considering two aspects that are tightly related to the proposed PMVT, i.e., the facial AU detection and vision transformer.

### 2.1. Methods for Facial AU Detection

Action units detection is a multi-label classification problem and has been studied for decades. Several AU detection methods have been proposed (Zhao et al., 2016; Li et al., 2017a,b; Shao et al., 2018; Li and Shan, 2021). To achieve higher AU detection accuracy, different hand-crafted features have been used to encode the characteristics of AUs, such as Histogram of Oriented Gradient (HOG), local binary pattern (LBP), Gabor (Benitez-Quiroz et al., 2016) etc. Recently, AU detection has achieved considerable improvements due to deep learning. Since AU corresponds to the movement of facial muscles, many methods detect the occurrence of AU based on location (Zhao et al., 2016; Li et al., 2017a,b; Shao et al., 2018). For example, Zhao et al. (2016) used a regionally connected convolutional layer and learned the region-specific convolutional filters from the sub-areas of the face. EAC-Net (Li et al., 2017b) and ROI (Li et al., 2017a) extracted AU features around the manually defined facial landmarks that are robust with respect to non-rigid shape changes. SEV-Net (Yang et al., 2021) utilized the AU semantic description as auxiliary information for AU detection. Jacob and Stenger (2021) used a transformer-based encoder to capture the relationships between AUs. However, these supervised methods rely on precisely annotated images and often overfit on a specific dataset as a result of insufficient training images.

Recently, weakly-supervised (Peng and Wang, 2018; Zhao et al., 2018) and self-supervised (Wiles et al., 2018; Li et al., 2019b, 2020; Lu et al., 2020) methods have attracted a lot of attention to mitigate the AU data scarcity issue. Weakly supervised methods typically use the incomplete AU annotations and learn AU classifiers from the prior knowledge between facial expression and facial AU (Peng and Wang, 2018). The self-supervised learning approaches usually adopt pseudo supervisory signals to learn facial AU representation without manual AU annotations (Li et al., 2019b, 2020; Lu et al., 2020). Among them, Lu et al. (2020) proposed a triplet ranking loss to learn AU representations *via* capturing the temporal AU consistency. Fab-Net (Wiles et al., 2018) was optimized to map a source facial frame to a target facial frame *via* estimating an optical flow field between the source and target frames. TCAE (Li et al., 2019b) was introduced to encode the pose-invariant facial AU representation *via* predicting separate displacements for pose and AU and using the cycle consistency in the feature and image domains simultaneously.

Our proposed PMVT differs from previous CNN-based or transformer-based (Jacob and Stenger, 2021) AU detection methods in two ways. One, PMVT does not rely on facial landmarks to crop the regional AU features. It is because the facial landmarks may suffer from considerable misalignments under severe facial poses. Under this condition, the encoded facial parts are not part-aligned and will lead to incorrect results. Two, PMVT is the multi-scale transformer-based and the self-attention and cross-attention mechanisms in PMVT can flexibly focus on a sequence of image fragments to encode the correlations among AUs. PMVT is potentially to obtain better facial AU detection performance than previous approaches. We will verify this in section 4.

### 2.2. Vision Transformer

Self-attention is capable of improving computer vision models due to its content-dependent interactions and parameter-independent scaling of the receptive fields, in contrast to previous parameter-dependent scaling and content-independent interactions of convolutions. Recently, self-attention-based transformer models have greatly facilitated research in machine translation and natural language processing tasks (Waswani et al., 2017). Transformer architecture has become the de-facto standard for a wide range of applications. The core intuition of the original transformer is to obtain self-attention by comparing a feature to all other features in the input sequence. In detail, features are first encoded to obtain a query (*Query*) and memory [(including key (*Key*) and value (*Value*)] embedding *via* linear projections. The product of *Query* with *Key* is used as the attention weight for *Value*. A position embedding is also introduced for each input token to remember the positional information which will be lost in the transformer, which is especially good at capturing long-range dependencies between tokens within an input sequence.

Inspired by this, many recent studies use transformers in various computer vision tasks (Dosovitskiy et al., 2020; Li et al., 2021). Among them, ViT (Dosovitskiy et al., 2020) introduces to view an image as a sequence of tokens and conduct image classification with a transformer encoder. To obtain the input patch features, ViT partition the input image into non-overlapping tokens with 16 × 16 spatial dimension and linearly project the tokens to match the encoder's input dimension. DeiT (Touvron et al., 2021) further proposes the data-efficient training and distillation for transformer-based image classification models. DETR (Carion et al., 2020) introduces an excellent object detection model based on the transformer, which considerably simplifies the traditional object detection pipeline and obtains comparable performances with prior CNN-based detectors. CrossViT (Chen et al., 2021) encodes small-patch and large-patch image tokens with two exclusive branches and these image tokens are then fused purely by a cross-attention mechanism. Subsequently, transformer models are further extended to other popular computer vision tasks such as segmentation (Jin et al., 2021), face recognition (Li et al., 2021), and 3D reconstruction (Lin et al., 2021). In this study, we extend CrossViT to facial AU detection and show its feasibility and superiority on two publicly available AU datasets.

## 3. Method

**Figure 2:**
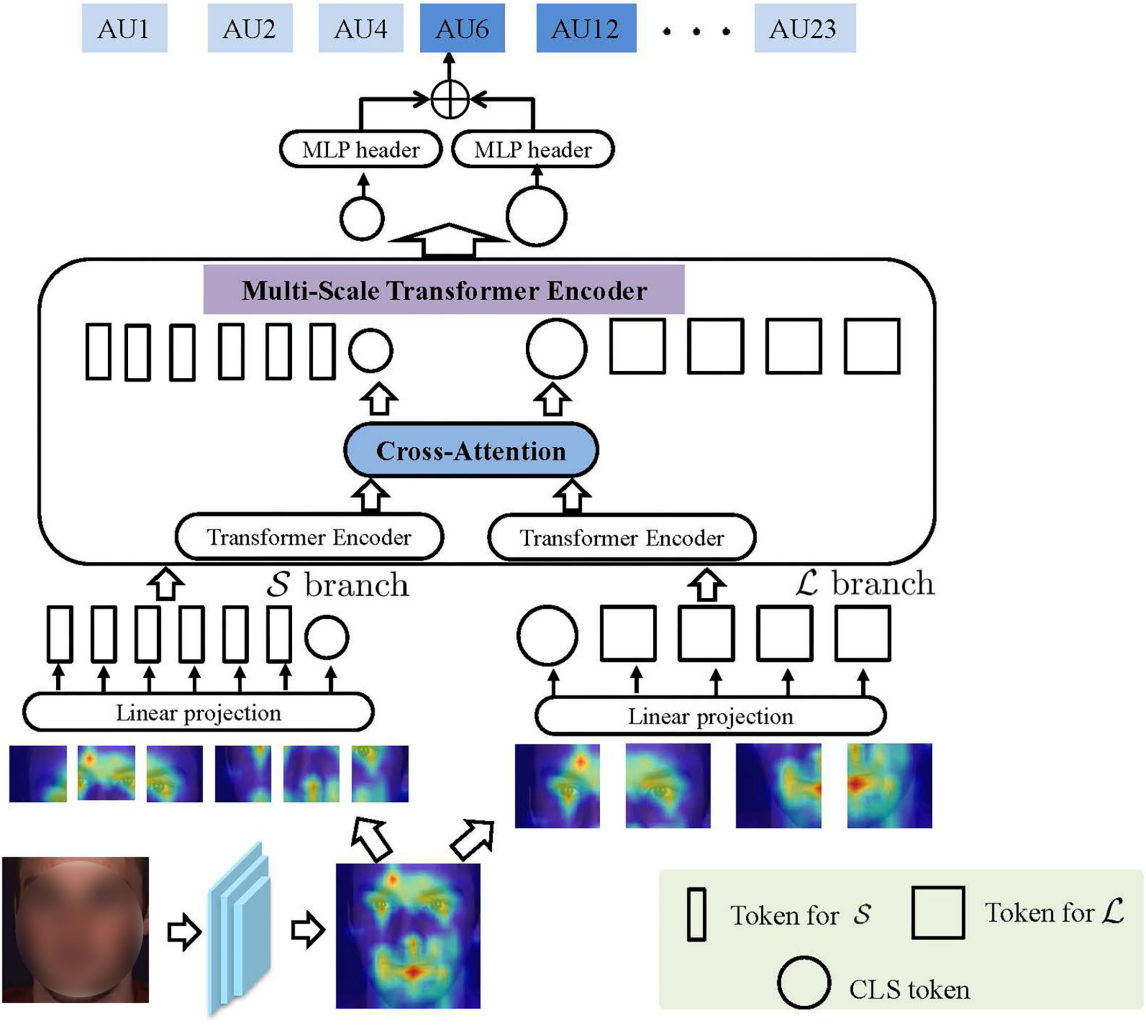
The main idea of the proposed progressive multi-scale vision transformer (PMVT). With the encoded convolutional feature map **X**_*con*_, PMVT uses *L* and *S* branch transformer encoders that each receives tokens with different resolutions as input. The two branches will be fused adaptively *via* cross-attention mechanism.

Figure 2 illustrates the main idea of the proposed PMVT. Given an input face, PMVT first extracts its convolutional feature maps *via* a commonly-used backbone network. Second, PMVT encodes the discriminative facial AU feature by the multi-scale transformer blocks. We will first review the traditional vision transformer and present our proposed PMVT afterward.

### 3.1. Revisiting Vision Transformer

We first revisit the critical components in ViT (Dosovitskiy et al., 2020) that mainly consist of image tokenization and several layers of the token encoder. Each encoder consists of two layers, i.e., multi-head self-attention (MSA) layer and feed-forward network (FFN) layer.

Traditional vision transformer typically receives a sequence of image patch embeddings as input. To obtain the token embeddings, ViT encodes the input image **X**∈ℝ^*H*×*W*×*C*^ into a set of flattened two-dimensional image patches: ${\text{X}}_{p}\;\in {\mathbb{R}}^{N\times P_{2}\times C}$. Among the mathematic symbols, *H* *W*, *C* denote the height, width, channel of the input image **X**. *P* means the spatial resolution of each image patch **X**_*p*_. After the image tokenization, we can obtain $N=\frac{H\times W}{P^{2}}$ patches that will be treated as the sequential input for the transformer. These image patches are then flattened and projected to embeddings with a size of *S*. Typically, ViT adds an extra class token that will be concatenated with the image embeddings, resulting in the input sequence with a size of ${\text{X}}_{t}\;\in {\mathbb{R}}^{(N+1)\times S}$. Finally, the class token will serve as the image representation that will be used for image classification. ViT uses a residual connection for each encoder. The computation in each encoder can be formulated as:


(1) $${\text{X}}_{t}^{\prime }=\text{LN}({\text{X}}_{t}+\text{MSA}({\text{X}}_{t})),$$

(2) $$\text{Y}=\text{LN}({\text{X}}_{t}^{\prime }++\text{FFN}({\text{X}}_{t}^{\prime })),$$

where **X**_*t*_ and **Y** denote the input and output of the encoder. ${\text{X}}_{t}^{\prime }$ is the output of the MSA layer. LN means layer normalization. MSA means multi-head self-attention which will be described next.

For the self-attention module in ViT, the sequential input tokens ${\text{X}}_{t}\in {\mathbb{R}}^{(N+1)\times S}$ are linearly transformed into *Query*, *Key*, *Value* spaces. Typically, *Query*∈ℝ^(*N*+1) × *S*^, *Key*∈ℝ^(*N*+1) × *S*^, *Value*∈ℝ^(*N*+1) × *S*^. Afterward, a weighted sum over all values in the sequential tokens is computed as,


(3) $$\begin{array}{r} \text{Attention}\left(Quey,Key,Value\right)=\text{softmax}\left(\frac{Query\times Key^{T}}{\sqrt{S}}\right)Value. \end{array}$$

Then a linear projection is conducted to the weighted values Attention(*Quey, Key, Value*). MSA is a natural extension of the single-head self-attention described above. MSA splits *Query*, *Key*, *Value* for *h* times and performs the self-attention mechanism in parallel, then maps their concatenated outputs *via* linear transformation. In addition to the MSA module, ViT exploits the FFN module to conduct dimension adjustment and non-linear transformation on each image token to enhance the representation ability of the transformed tokens.

### 3.2. Progressive Multi-Scale Transformer

The direct tokenization of input images into large patches in ViT has been found to show its limitations (Yuan et al., 2021). On the one hand, it is difficult to perceive the important low-level characteristics (e.g., edges, colors, corners) in images; On the other hand, large CNN kernels for the image tokenization contain too many trainable parameters and are often difficult to optimize, and thus, ViT requires much more training samples. This is particularly impartial for facial AU detection. As AU annotation is time-consuming, cumbersome, and error-prone. Currently, the publicly available AU datasets merely contain limited facial images. To cope with this issue, we exploit the popular ResNet-based backbone to encode the input facial image **X** to obtain the convolutional feature map **X**_*con*_ = *F*(**X**), where *F* means the neural operation in the backbone network.

To obtain multi-scale tokens from **X**_*con*_, we use two separate branch transformer encoder that each receives tokens with different resolution as input. We illustrate the main idea of our proposed PMVT in Figure 2. Mathematically speaking, let us denote the two branches as L and S, respectively. In PMVT, the L branch uses coarse-grained token as input while the S branch directly operates at a much more fine-grained token. Both branches are adaptively fused *K* times *via* a cross-attention mechanism. Finally, PMVT exploits the *CLS* token of the L and S branches for facial AU detection. For each token within each branch, PMVT introduces a trainable position embedding. Note that we can use multiple multi-scale transformer encoders (MST) or perform cross-attention times within each MST. We will analyze the performance variations in section 4.2.1.

**Figure 3:**
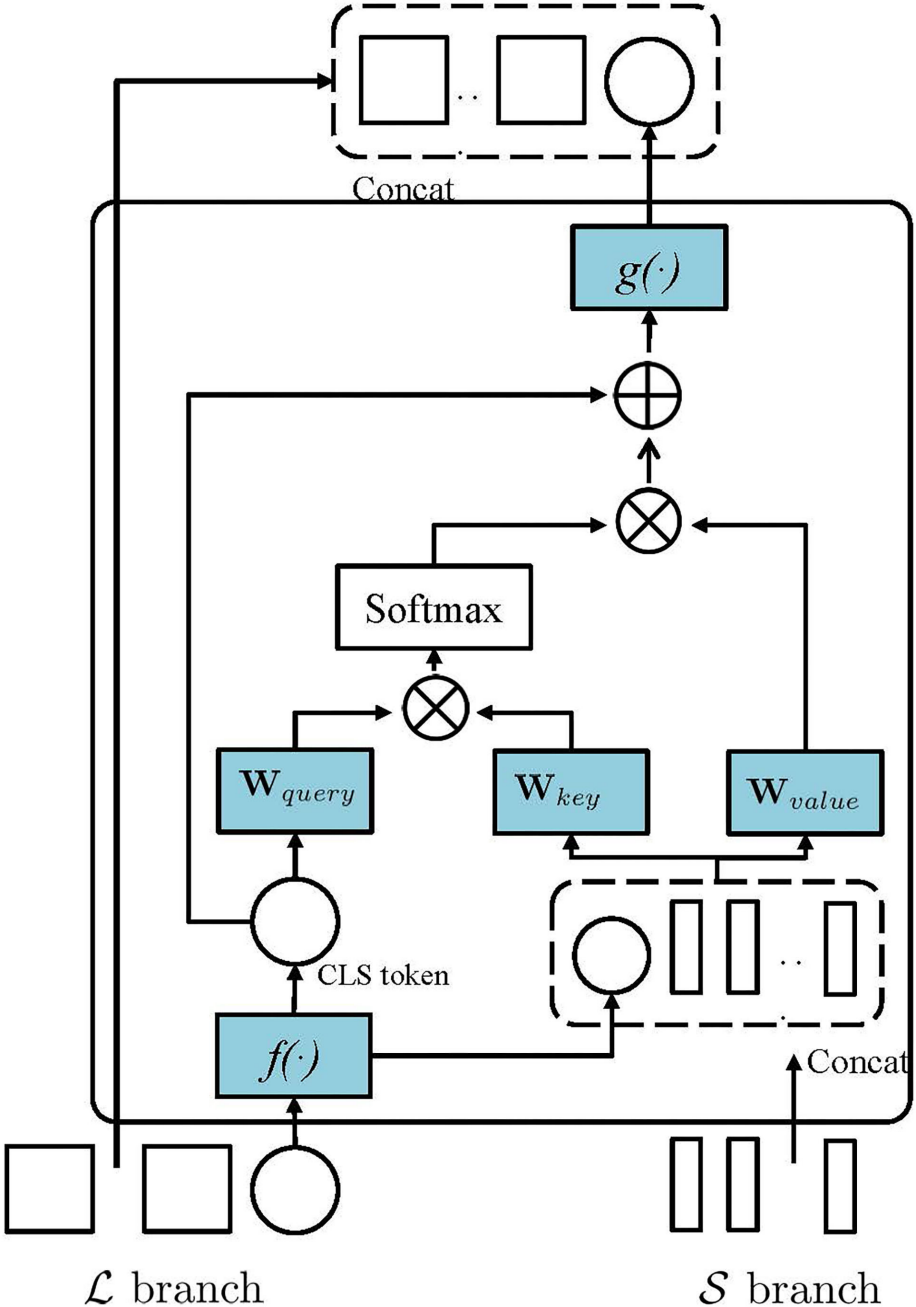
The main idea of the cross-attention in PMVT. In this study, we show that PMVT utilizes the classification (*CLS*) token at the *L* branch as an agent to exchange semantic AU information among the patch tokens from the *S* branch. PMVT can also use the *CLS* token at *S* to absorb information among the tokens from the *L* branch.

Figure 3 illustrates the cross-attention mechanism in PMVT. To effectively fuse the multi-scale AU features, PMVT utilizes the *CLS* token at each branch (e.g., L branch) as an agent to exchange semantic AU information among the patch tokens from the other branch (e.g., S branch) and then project the *CLS* token back to its own branch (e.g., L branch). Such operation is reasonable because the *CLS* token in L or S branch already learns semantic features among all patch tokens in its own branch. Thus, interacting with the patch tokens at the other branch can absorb more semantic AU information at a different scale. We hypothesize such cross-attention mechanism will help learn discriminative AU features as different AU usually have different appearance scopes and there exist correlations among the facial AUs. The multi-scale features will help encode AUs more precisely and PMVT will encode the AU correlations with the self-/cross-attention mechanism.

Take L for example to show the cross-attention mechanism in PMVT. Specially, PMVT uses the *CLS* token ${\text{X}}_{cls}^{l}$ from the L branch and patch tokens the ${\text{X}}_{i}^{s}$ from S branch for feature fusing. PMVT uses ${\text{X}}_{cls}^{l}$ to obtain a *query* and use ${\text{X}}_{i}^{s}$ to obtain the *key* and *value*. The *query*, *key*, *value* will be transformed into a weighted sum overall values in the sequential tokens as that in Equation (3). Notably, such a cross-attention mechanism is similar to self-attention except that the *query* is obtained from the *CLS* token in another transformer branch. In Figure 3, *f*(.) and *g*(.) mean linear projections that aim the alignment of the feature dimension. We will evaluate the effectiveness of the proposed PMVT in the next section.

### 3.3. Training Objective

We utilize the multi-label sigmoid cross-entropy loss for training the facial AU detection model in PMVT, which can be formulated as:


(4) $$\begin{array}{r} L^{AU}=-\overset{J}{\underset{j}{\sum }}z^{j}\log{ẑ}^{j}+\left(1-z^{j}\right)\log\left(1-{ẑ}^{j}\right), \end{array}$$

where *J* denotes the number of facial AUs. *z*^*j*^ denotes the *j*-th ground truth AU annotation of the input AU sample. ẑ^*j*^ means the predicted AU score. *z*_*i*_∈{0, 1} denotes the annotation with respect to the *i*th AU. 1 means the AU is active, 0 means inactive.

## 4. Experiment

### 4.1. Implementation Details

We adopted ResNet-34 (He et al., 2016) as the backbone network for PMVT due to its elegant network structure and excellent performance in image classification. We chose the output of the third stage as the convolutional feature maps: ${\text{X}}_{con}\in {\mathbb{R}}^{14\times 14\times 512}$. For the *L* branch, the token size is set as *N* = 5 × 5 *via* adaptative pooling operation. For the *S* branch, the token size is set as *N* = 14 × 14. The pre-trained model based on the ImageNet dataset was used for initializing the backbone network. For the transformer part, we use one layer of transformer encoder that consists of two-layer cross-attention. We exploited a batch-based stochastic gradient descent method to optimize the proposed PMVT. During the training process, we set the batch size as 64 and the initial learning rate as 0.002. The momentum was set as 0.9 and the weight decay was set as 0.0005.

#### 4.1.1. Datasets

For AU detection, we adopted BP4D (Zhang et al., 2013) and DISFA (Mavadati et al., 2013) datasets. Among them, BP4D is a spontaneous FACS dataset that consists of 328 videos for 41 subjects (18 men and 23 women). Eight different tasks are evaluated on a total of 41 participants, and their spontaneous facial expression variations were recorded in several videos. Each participant subject is involved in eight sessions, and their spontaneous facial expressions were captured in both 2D and 3D videos. A total of 12 AUs were annotated for the 328 videos, and there are approximately 1,40,000 frames with AU annotations. DISFA contains 27 participants that consists of 12 women and 15 men. Each subject is asked to watch a 4-min video to elicit their facial AUs. The facial AUs are annotated with intensities from 0 to 5. In our experiments, we obtained nearly 1,30,000 AU-annotated images in the DISFA dataset by considering the images with intensities greater than 1 as active. For BP4D and DISFA datasets, the images are split into 3-fold in a subject-independent manner. Based on the datasets, we conducted 3-fold cross-validation. We adopted 12 AUs in BP4D and 8 AUs in DISFA dataset for evaluation. For the DISFA dataset, we leveraged the model trained on BP4D to initialize the backbone network, following the same experimental setting of Li et al. (2017b).

#### 4.1.2. Evaluation Metric

We adopted F1-score ($F1=\frac{2RP}{R+P}$) to evaluate the performance of the proposed AU detection method, where *R* and *P*, respectively, denote recall and precision. We additionally calculated the average F1-score over all AUs (AVE) to quantitatively evaluate the overall facial AU detection performance. We show the AU detection results as *F*1 × 100.

### 4.2. Experimental Results

We compare the proposed with the state-of-the-art facial AU detection approaches, including DRML (Zhao et al., 2016), EAC-Net (Li et al., 2017b), ROI (Li et al., 2017a), JAA-Net (Shao et al., 2018), OFS-CNN (Han et al., 2018), DSIN (Corneanu et al., 2018), TCAE (Li et al., 2019b), TAE (Li et al., 2020), SRERL (Li et al., 2019a), ARL (Shao et al., 2019), SEV-Net (Yang et al., 2021), and FAUT (Jacob and Stenger, 2021). Among them, most of the AU methods (Li et al., 2017a, 2019a; Corneanu et al., 2018; Shao et al., 2018) manually crop the local facial regions to learn the AU-specific representations with exclusive CNN branches. TAE (Li et al., 2020) utilize unlabeled videos that consist of approximately 7,000 subjects to encode the AU-discriminative representation without AU annotations. SEV-Net (Yang et al., 2021) introduce the auxiliary semantic word embedding and visual feature for AU detection. FAUT (Jacob and Stenger, 2021) introduce an AU correlation network based on a transformer architecture to perceive the relationships between different AU in an end-to-end manner.

Table 1 shows the AU detection results of our method and studies works on the BP4D dataset. Our PMVT achieves comparable AU detection accuracy with the best state-of-the-art AU detection methods in the average F1 score. Compared with other methods, PMVT obtains consistent improvements in the average accuracy (+14.6% over DRML, +7.0% over EAC-Net, +6.5% over ROI, +2.9% over JAA-Net, +4.0% over DSIN, +6.8% over TCAE, +2.6% over TAE). The benefits of our proposed PMVT over other methods can be explained in 2-fold. First, PMVT explicitly introduces transformer modules in the network structure. The self-attention mechanism in the transformer modules is capable of perceiving the local to global interactions between different facial AUs. Second, we use multi-scale features to better encode the regional features of the facial AUs, as different AUs have different appearance scopes. The cross-attention mechanism between the multi-scale features is beneficial for learning discriminative facial AU representations. Table 2 shows the quantitative facial AU detection results of our PMVT and other methods on the DISFA dataset. PMVT achieves the second-best AU detection accuracy compared with all the state-of-the-art AU detection methods in the average F1 score. In detail, PMVT outperforms EAC-Net, JAA-Net, OFS-CNN, TCAE, TAE, SRERL, ARL, and SEV-Net with +12.4%, +4.9%, +9.5%, +7.3%, +15.9%, +9.4%, +5.0%, +2.2%, and +2.1% improvements in the average F1 scores. The consistent improvements over other methods on the two popular datasets verify the feasibility and superiority of our proposed PVMT. We will carry out an ablation study to investigate the contribution of the self-/cross-attention in PVMT and illustrate visualization results in the next section.

**Table 1:** Action unit (AU) detection performance of our proposed progressive multi-scale vision transformer (PMVT) and state-of-the-art methods on the BP4D dataset.

**Methods**	**AU1**	**AU2**	**AU4**	**AU6**	**AU7**	**AU10**	**AU12**	**AU14**	**AU15**	**AU17**	**AU23**	**AU24**	**AVE**
LSVM (Fan et al., 2008)	23.2	22.8	23.1	27.2	47.1	77.2	63.7	64.3	18.4	33.0	19.4	20.7	35.3
DRML (Zhao et al., 2016)	36.4	41.8	43.0	55.0	67.0	66.3	65.8	54.1	33.2	48.0	31.7	30.0	48.3
EAC-Net (Li et al., 2017b)	39.0	35.2	48.6	76.1	72.9	81.9	86.2	58.8	37.5	59.1	35.9	35.8	55.9
ROI (Li et al., 2017a)	36.2	31.6	43.4	77.1	73.7	85.0	87.0	62.6	45.7	58.0	38.3	37.4	56.4
JAA-Net (Shao et al., 2018)	47.2	44.0	54.9	77.5	74.6	84.0	86.9	61.9	43.6	60.3	42.7	41.9	60.0
DSIN (Corneanu et al., 2018)	51.7	40.4	56.0	76.1	73.5	79.9	85.4	62.7	37.3	62.9	38.8	41.6	58.9
TCAE (Li et al., 2019b)	43.1	32.2	44.4	75.1	70.5	80.8	85.5	61.8	34.7	58.5	37.2	48.7	56.1
TAE (Li et al., 2020)	47.0	45.9	50.9	74.7	72.0	82.4	85.6	62.3	48.1	62.3	45.9	46.3	60.3
SRERL (Li et al., 2019a)	46.9	45.3	55.6	77.1	78.4	83.5	**87.6**	63.9	52.2	**63.9**	47.1	53.3	62.9
ARL (Shao et al., 2019)	45.8	39.8	55.1	75.7	77.2	82.3	86.6	58.8	47.6	62.1	47.4	55.4	61.1
FAUT (Jacob and Stenger, 2021)	51.7	49.3	**61.0**	77.8	**79.5**	82.9	86.3	**67.6**	51.9	63.0	43.7	**56.3**	**64.2**
SEV-Net (Yang et al., 2021)	58.2	**50.4**	58.3	81.9	73.9	**87.8**	87.5	61.6	52.6	62.2	44.6	47.6	63.9
**PMVT (Ours)**	**59.3**	43.0	59.3	**82.3**	73.6	82.6	86.1	57.6	**53.0**	60.2	**47.9**	50.6	62.9

*The highest values are illustrated in Bold format*.

**Table 2:** Action unit detection performance of our proposed PMVT and state-of-the-art methods on the DISFA dataset.

**Methods**	**AU1**	**AU2**	**AU4**	**AU6**	**AU9**	**AU12**	**AU25**	**AU26**	**ave**
DRML (Zhao et al., 2016)	17.3	17.7	37.4	29.0	10.7	37.7	38.5	20.1	26.7
EAC-Net (Li et al., 2017b)	41.5	26.4	66.4	50.7	**80.5**	**89.3**	88.9	15.6	48.5
JAA-Net (Shao et al., 2018)	43.7	46.2	56.0	41.4	44.7	69.6	88.3	58.4	56.0
OFS-CNN (Han et al., 2018)	43.7	40.0	67.2	59.0	49.7	75.8	72.4	54.8	51.4
DSIN (Corneanu et al., 2018)	42.4	39.0	**68.4**	28.6	46.8	70.8	90.4	42.2	53.6
TCAE (Li et al., 2019b)	15.1	15.2	50.5	48.7	23.3	72.1	82.1	52.9	45.0
TAE (Li et al., 2020)	21.4	19.6	64.5	46.8	44.0	73.2	85.1	55.3	51.5
SRERL (Li et al., 2019a)	45.7	47.8	59.6	47.1	45.6	73.5	84.3	43.6	55.9
FAUT (Jacob and Stenger, 2021)	46.1	48.6	72.8	**56.7**	50.0	72.1	90.8	55.4	**61.5**
ARL (Shao et al., 2019)	43.9	42.1	63.6	41.8	40.0	76.2	95.2	**66.8**	58.7
SEV-Net (Yang et al., 2021)	**55.3**	53.1	61.5	53.6	38.2	71.6	95.7	41.5	58.8
**PMVT (Ours)**	50.0	**54.3**	63.2	55.6	40.0	72.2	**95.9**	56.3	60.9

*The highest values are illustrated in Bold format*.

#### 4.2.1. Ablation Study

We illustrate the ablation study experimental results in Table 3. In Table 3, we show the AU detection performance variations with different cross-attention layers (*CL* = 1, 2, 3) in the multi-scale transformer encoder and with different layers of multi-scale transformer encoders (*MS* = 1, 2, 3).

**Table 3:** Ablation studies on the BP4D and DISFA datasets.

**Methods**	**BP4D**	**DISFA**
CL=1	60.7	56.3
CL=2	62.9	60.9
CL=3	59.5	55.8
MS=1	62.9	60.9
MS=2	59.8	58.1
MS=3	55.0	51.1

As shown in Table 3, PMVT shows its best AU detection performance with *CL* = 2 and *MS* = 1. It means PMVT merely contains one layer of the multi-scale transformer encoder, and the encoder contains two layers of cross-attention. With more MST encoders, PMVT will contain too many trainable parameters and will suffer from insufficient training images. With *CL* = 1 or *CL* = 3, PMVT shows degraded AU detection performance, and it suggests that information fusion should be performed twice to achieve the discriminative AU representations.

**Figure 4:**
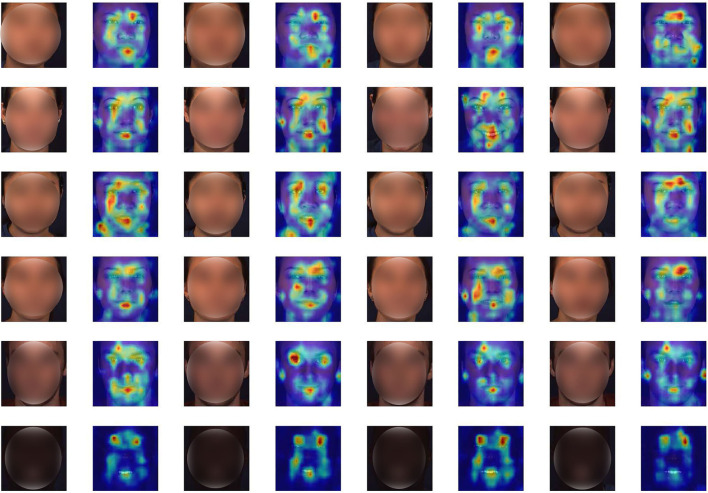
Attention maps of some representative faces. We illustrate a subject with different facial expressions in each row. It is obvious that the proposed PMVT is capable of focusing on the most silent parts for facial AU detection. Deep red denotes high activation, better viewed in color and zoom in.

We additionally show the attention maps of PMVT on some randomly sampled faces in Figure 4. The visualization results show the benefits of the proposed PMVT for robust facial AU detection. It is obvious that PVMT shows consistent activation maps for each face under different races, expressions, lightings, and identities. For example, the third face in the second row is annotated with active AU1 (inner brow raiser), AU2 (outer brow raiser), AU6 (cheek raiser), AU7 (inner brow raiser), AU10 (inner brow raiser), and AU12 (inner brow raiser). The second face in the third row is annotated with active AU1 (inner brow raiser), AU10 (inner brow raiser), AU12 (inner brow raiser), and AU15 (lip corner depressor). The first face in the fourth row is annotated with active AU7 (inner brow raiser) and AU14 (dimpler). The attention maps of these faces are in line and consistent with the annotated AUs. The visualization maps in Figure 4 show the generalization ability and feasibility of our proposed PVMT.

## 5. Conclusions

In this study, we propose a PMVT to perceive the complex relationships among different AUs in an end-to-end data-driven manner. PMVT is based on the multi-scale self-/cross-attention mechanism that can flexibly focus on sequential image patches to effectively encode the discriminative AU representation and perceive the correlations among different facial AUs. Compared with previous facial AU detection methods, PMVT obtains comparable AU detection performance. Visualization results show the superiority and feasibility of our proposed PMVT. For future study, we will explore utilizing PMVT for more affective computing tasks, such as facial expression recognition, AU density estimation.

## Data Availability Statement

The original contributions presented in the study are included in the article/supplementary material, further inquiries can be directed to the corresponding author/s.

## Author Contributions

CW and ZW cooperatively completed the method design and experiment parts. CW wrote all the sections of the manuscript. ZW carried out the experiments and gave the detailed analysis. Both the two authors have carefully read, polished, and approved the final manuscript.

## Conflict of Interest

The authors declare that the research was conducted in the absence of any commercial or financial relationships that could be construed as a potential conflict of interest.

## Publisher's Note

All claims expressed in this article are solely those of the authors and do not necessarily represent those of their affiliated organizations, or those of the publisher, the editors and the reviewers. Any product that may be evaluated in this article, or claim that may be made by its manufacturer, is not guaranteed or endorsed by the publisher.

## References

[B1] Bartlett M. S. Littlewort G. Fasel I. Movellan J. R. (2003). “Real time face detection and facial expression recognition: development and applications to human computer interaction,” in 2003 Conference on Computer Vision and Pattern Recognition Workshop, Vol. 5 (Madison, WI: IEEE), 53–53.

[B2] Benitez-Quiroz C. F. Srinivasan R. Martinez A. M. (2016). “Emotionet: An accurate, real-time algorithm for the automatic annotation of a million facial expressions in the wild,” in Proceedings of the IEEE conference on computer vision and pattern recognition (CVPR), 5562–5570.

[B3] Carion N. Massa F. Synnaeve G. Usunier N. Kirillov A. Zagoruyko S. (2020). “End-to-end object detection with transformers,” in European Conference on Computer Vision (Glasgow: Springer), 213–229.

[B4] Chen C.-F. Fan Q. Panda R. (2021). Crossvit: cross-attention multi-scale vision transformer for image classification. arXiv preprint arXiv:2103.14899.

[B5] Corneanu C. Madadi M. Escalera S. (2018). “Deep structure inference network for facial action unit recognition,” in ECCV (Munich), 298–313.

[B6] Dosovitskiy A. Beyer L. Kolesnikov A. Weissenborn D. Zhai X. Unterthiner T. . (2020). An image is worth 16x16 words: transformers for image recognition at scale. arXiv preprint arXiv:2010.11929.

[B7] Ekman P. Friesen W. V. (1978). Manual for the Facial Action Coding System. Consulting Psychologists Press.

[B8] Fan R.-E. Chang K.-W. Hsieh C.-J. Wang X.-R. Lin C.-J. (2008). Liblinear: a library for large linear classification. J. Mach. Learn. Res. 9, 1871–1874. Available online at: https://dl.acm.org/citation.cfm?id=144279421207929

[B9] Fang Y. Gao S. Li J. Luo W. He L. Hu B. (2020). Multi-level feature fusion based locality-constrained spatial transformer network for video crowd counting. Neurocomputing 392, 98–107. 10.1016/j.neucom.2020.01.087

[B10] Girdhar R. Carreira J. Doersch C. Zisserman A. (2019). “Video action transformer network,” in Proceedings of the IEEE/CVF Conference on Computer Vision and Pattern Recognition, (Long Beach, CA: IEEE), 244–253.

[B11] Han S. Meng Z. Li Z. O'Reilly J. Cai J. Wang X. . (2018). “Optimizing filter size in convolutional neural networks for facial action unit recognition,” in Proceedings of the IEEE Conference on Computer Vision and Pattern Recognition (Salt Lake City, UT), 5070–5078.

[B12] He K. Zhang X. Ren S. Sun J. (2016). “Deep residual learning for image recognition,” in Proceedings of the IEEE Conference on Computer Vision and Pattern Recognition, 770–778.32166560

[B13] Jacob G. M. Stenger B. (2021). “Facial action unit detection with transformers,” in Proceedings of the IEEE/CVF Conference on Computer Vision and Pattern Recognition, 7680–7689.

[B14] Jin Y. Han D. Ko H. (2021). Trseg: transformer for semantic segmentation. Pattern Recognit. Lett. 148, 29–35. 10.1016/j.patrec.2021.04.024

[B15] Li G. Zhu X. Zeng Y. Wang Q. Lin L. (2019a). “Semantic relationships guided representation learning for facial action unit recognition,” in AAAI, vol. 33, 8594–8601.

[B16] Li W. Abtahi F. Zhu Z. (2017a). “Action unit detection with region adaptation, multi-labeling learning and optimal temporal fusing,” in CVPR (Honolulu, HI: IEEE).

[B17] Li W. Abtahi F. Zhu Z. Yin L. (2017b). “Eac-net: a region-based deep enhancing and cropping approach for facial action unit detection,” in FG (Washington, DC). 10.1109/TPAMI.2018.2791608 29994168

[B18] Li Y. Shan S. (2021). Meta auxiliary learning for facial action unit detection. arXiv preprint arXiv:2105.06620.

[B19] Li Y. Sun Y. Cui Z. Shan S. Yang J. (2021). Learning fair face representation with progressive cross transformer. arXiv preprint arXiv:2108.04983.

[B20] Li Y. Zeng J. Shan S. (2020). Learning representations for facial actions from unlabeled videos. IEEE Trans. Pattern Anal. Mach. Intell. 44, 302–317. 10.1109/TPAMI.2020.3011063 32750828

[B21] Li Y. Zeng J. Shan S. Chen X. (2018a). Occlusion aware facial expression recognition using cnn with attention mechanism. IEEE Trans. Image Process. 28, 2439–2450. 10.1109/TIP.2018.2886767 30571627

[B22] Li Y. Zeng J. Shan S. Chen X. (2018b). “Patch-gated cnn for occlusion-aware facial expression recognition,” in 2018 24th International Conference on Pattern Recognition (ICPR) (Beijing: IEEE), 2209–2214.

[B23] Li Y. Zeng J. Shan S. Chen X. (2019b). “Self-supervised representation learning from videos for facial action unit detection,” in Proceedings of the IEEE/CVF Conference on Computer Vision and Pattern Recognition (Long Beach, CA), 10924–10933.

[B24] Lin K. Wang L. Liu Z. (2021). “End-to-end human pose and mesh reconstruction with transformers,” in Proceedings of the IEEE/CVF Conference on Computer Vision and Pattern Recognition (Long Beach, CA), 1954–1963.

[B25] Lu L. Tavabi L. Soleymani M. (2020). “Self-supervised learning for facial action unit recognition through temporal consistency,” in Proceedings of the British Machine Vision Conference (BMVC). BMVA Press.

[B26] Mavadati S. M. Mahoor M. H. Bartlett K. Trinh P. Cohn J. F. (2013). Disfa: a spontaneous facial action intensity database. IEEE Trans. Affect. Comput. 4, 151–160. 10.1109/T-AFFC.2013.427295638

[B27] Peng G. Wang S. (2018). “Weakly supervised facial action unit recognition through adversarial training,” in CVPR (Salt Lake City, UT), 2188–2196.

[B28] Shao Z. Liu Z. Cai J. Ma L. (2018). “Deep adaptive attention for joint facial action unit detection and face alignment,” in ECCV Munich.

[B29] Shao Z. Liu Z. Cai J. Wu Y. Ma L. (2019). Facial action unit detection using attention and relation learning. IEEE Trans. Affect. Comput. 10.1109/TAFFC.2019.294863527295638

[B30] Touvron H. Cord M. Douze M. Massa F. Sablayrolles A. Jegou H. (2021).“Training data-efficient image transformers and distillation through attention,” in Proceedings of the 38th International Conference on Machine Learning (ICML), 10347–10357.

[B31] Waswani A. Shazeer N. Parmar N. Uszkoreit J. Jones L. Gomez A. . (2017).“Attention is all you need,” in Proceedings of the Conference on Neural Information Processing Systems (Long Beach, CA), 1–11.

[B32] Wiles O. Koepke A. Zisserman A. (2018). Self-supervised learning of a facial attribute embedding from video. arXiv preprint arXiv:1808.06882.

[B33] Yang H. Yin L. Zhou Y. Gu J. (2021). “Exploiting semantic embedding and visual feature for facial action unit detection,” in Proceedings of the IEEE/CVF Conference on Computer Vision and Pattern Recognition (Nashville, TN), 10482–10491.

[B34] Yuan K. Guo S. Liu Z. Zhou A. Yu F. Wu W. (2021). Incorporating convolution designs into visual transformers. arXiv preprint arXiv:2103.11816.

[B35] Zafar Z. Khan N. A. (2014). “Pain intensity evaluation through facial action units,” in 2014 22nd International Conference on Pattern Recognition (Stockholm:IEEE), 4696–4701.

[B36] Zhang X. Yin L. Cohn J. F. Canavan S. Reale M. Horowitz A. Liu P. (2013). “A high-resolution spontaneous 3d dynamic facial expression database,” in FG (Shanghai: IEEE).

[B37] Zhao K. Chu W.-S. Martinez A. M. (2018). “Learning facial action units from web images with scalable weakly supervised clustering,” in CVPR (Salt Lake City, UT), 2090–2099. 10.1109/CVPR.2018.00223 PMC6594709 31244515

[B38] Zhao K. Chu W.-S. Zhang H. (2016). “Deep region and multi-label learning for facial action unit detection,” in CVPR (Las Vegas, NV), 3391–3399.

